# Should carbon removal be treated as waste management? Lessons from the cultural history of waste

**DOI:** 10.1098/rsfs.2020.0010

**Published:** 2020-08-14

**Authors:** Holly Jean Buck

**Affiliations:** UCLA Institute of the Environment and Sustainability and UCLA School of Law, Los Angeles, CA, USA

**Keywords:** negative emissions, carbon utilization, carbon dioxide removal, waste management, chemurgy

## Abstract

Carbon dioxide is a waste product of combusting fossil fuels, and its accumulation in the atmosphere presents a planetary hazard. Carbon dioxide is also managed and used as a resource. Emerging technologies like direct air capture present the opportunity to reclaim and re-use wasted carbon, and actors in industry and policy are increasingly understanding carbon capture, utilization and storage as a waste management process. What is the value, and the danger, of conceptualizing CO_2_ as a waste to be managed? This paper looks at the historical evolution of solid and liquid waste regimes to draw lessons for the future evolution of a gaseous waste regime. It finds that social decisions to clean up solid and liquid waste were driven by both culture and industry. Views of recycling and sanitation did not evolve smoothly, with recycling falling in and out of favour, and sanitation experiencing conflict between public and private actors. An earlier attempt to revalue waste as part of a circular economy—the 1930s scientific and industrial field of chemurgy—failed to become a durable term and movement. These experiences hold important takeaways for negative emissions technologies and carbon removal policy: technocratic ideas about resource management may not take hold without a broader popular movement, as in the case of chemurgy, but value change and technology development can support each other, as in the case of wastewater infrastructure. Scientists and carbon removal policy advocates have an opportunity to contextualize CO_2_ waste management within the struggles and goals of the larger circular economy project, and to focus simultaneously on both waste production and waste disposal.

## 1. Introduction

Carbon dioxide removal (CDR) can be thought of as waste management on a gigaton scale. The IPCC's assessment that limiting warming to 1.5°C relies on 100–1000 gigatons of CDR [1] provokes the conversation of what sinks might be found to dispose of all that removed carbon. Anthropologist Mary Douglas famously said that ‘dirt is matter out of place’ [2]. Carbon dioxide in the atmosphere can also be viewed as carbon that is simply ‘out of place’. With the advent of direct air capture and other technologies, it is now possible to think of moving that carbon in the atmosphere elsewhere, such as underground for deep storage.

At the same time, emerging talk about the ‘carbon-to-value’ economy, as well as legislation supporting CCUS (carbon capture, utilization and storage) rather than simply carbon capture and storage (CCS), frames carbon dioxide as an emergent resource to be used in novel products. CCUS is also used in conjunction with enhanced oil recovery, which is an oil extraction process that at present relies largely on natural deposits of CO_2_ rather than anthropogenically sourced CO_2_. CO_2_ emissions can be used to produce a variety of chemicals, useable in materials like plastics or in liquid fuels [3]. Notably, it has been estimated that the chemical conversion of CO_2_ is unlikely to account for more than 1% of the mitigation challenge, and even scaling up enhanced oil recovery with CCS would only account for 4–8% [4]. But this has not limited interest in the approach, and as Bruhn and colleagues observe, CCUS and CCS are commingled in political contexts [3]. For example, a bipartisan piece of legislation introduced in 2019 in the US Congress as the ‘Utilizing Significant Emissions with Innovative Technologies’ or USE IT Act calls for an inventory of ‘current or emerging activities that transform captured carbon dioxide into a product of commercial value, or as an input to products of commercial value’ [5]. Fossil fuel companies, some of which have decades of experience with enhanced oil recovery using carbon dioxide, are also interested in carbon utilization (see [6]) and the competitive advantage of providing fossil fuels with lower carbon intensities. As stated by Saudi Aramco, ‘Capturing carbon has been used for decades as a way to help improve the quality of natural gas, but by pioneering new technologies we can now remove and sequester CO_2_ indefinitely. Moreover, we can now add value to what has always been considered a waste product, by turning CO_2_ into marketable industrial and commercial products’ [7]. In the frame of CCUS, CO_2_ is not just a waste, but a useful waste.

On the level of big-picture conceptual frame, waste management is an increasingly powerful analogy for CDR. But it is also more than just an analogy: many forms of carbon removal are literally integrated with other forms of waste management. Bioenergy with carbon capture and storage, for example, can involve combusting not just crop residues but municipal solid waste; biochar can also be made with waste. Both these techniques could become part of the bioeconomy. For example, a recent analysis of how California could achieve net-zero emissions by Lawrence Livermore National Laboratory relied on a reimagining of California's municipal, agricultural and forestry waste flows as the key pillar, estimating that 84 million tons of CO_2_ per year could be removed by converting waste biomass to fuels and storing the CO_2_ [8]. In this instance, waste management is not a metaphor for carbon removal, but the central mechanism of it.

Waste is a complex and multivalent term. It is derived from the old French *vastum*, with resonances of an empty or desolate land. It can be an externality, a commodity and a livelihood [9]. Sabine Barles points to three different types of vocabularies that have been used to describe waste [10]. The first involves themes of loss and uselessness: *déchet* (French), refuse and garbage, *residuo* (Spanish), *Abfall* (German). The second involves terms that involve uncleanliness or repulsion: *immondice* (French), *immondizia* (Italian), *ordure* (French). Third, there are terms relating to materials that make up the waste: *boues* (French), *spazzatura* (Italian), *Müll* and *Schmutz* (German), *rubbish* [10, p. 200]. Clearly, there are multiple ways to view waste, but we can focus on two main views of ‘waste’ that are relevant for CDR practices.

The first view of CO_2_ as waste is something that is dangerous, hazardous or icky; that which must be managed and disposed of safely. This view can easily be applied to geologic CO_2_ disposal. When it comes to geological carbon capture and storage, the technologies employed draw upon a similar knowledge base to other forms of subsurface disposal in the fossil fuel and hazardous waste disposal industry: from an engineering point of view, carbon capture and disposal is similar to acid gas disposal at more than 70 sites in North America, and other fluid-waste disposal operations, though on a large scale [11, p. 25]. In fact, ‘disposal’ is an accurate term for CCS, but ‘storage’ and ‘sequestration’ have been preferred by the industry, perhaps to avoid associations with waste disposal and concurrent environmental justice and public acceptance concerns. CO_2_ disposal in this sense also has parallels with radioactive waste, in that it involves looking for a space with reasonable tectonic stability, a natural barrier against migration, and containment over long time-scales (centuries to millennia for CO_2_, much longer for radioactive waste), which brings up issues around liability, the willingness of communities to accept this waste, and intergenerational equity [12]. We can call this view of CO_2_ as waste the ‘permanent disposal paradigm’. It aligns with CCS.

The second view is the circular economy view of waste as a resource: something that is failed to be used. The modern incarnation of this view has conceptual roots in the environmental paradigm of the 1960s and 1970s, when we began to view the Earth as a finite planet with limits, and hence waste became a problem for spaceship Earth. Studies of industrial ecology, life cycle assessment and social metabolism have contributed to our understanding of this. Blomsma & Brennan [13] trace the umbrella concept of the circular economy, noting that from around 1985 onward, there was excitement about viewing waste as a source of value, with the wider discussion around sustainable development surfacing during this time. Since the mid-2000s, Zero Waste and Circular Economy programmes have been popular around the world, and this discourse has also been connected with climate change, as O'Neill explores [14]. The circular economy is ‘restorative and regenerative by design’, as the Ellen MacArthur Foundation puts it, underpinned by a transition to renewable energy [14]. The circular model is not simply about dealing with waste, but producer-led transformation involving both industrial symbiosis and extended product life (see [15]). We can call this view of waste the ‘circular paradigm’. Waste is eliminated through utilization. The discourses of carbon management, carbon-to-value, etc. draw heavily on this circular paradigm, which aligns with CCUS.

In many respects, the general analogy of waste management in both these permanent disposal and circular forms serves useful purposes. It identifies a social challenge, waste management, which societies have dealt with unevenly and insufficiently, but with which we have a lot of experience. Moving to treat CO_2_ as waste suggests a progression of modernity and progress, creating a parallel narrative with how modern sanitation and recycling evolved: first, we observed the problem. Then policy action was taken and cultural norms shifted, in a concurrent process, so that people were willing to pay to clean up the problem. Klaus Lackner and Christophe Jospe detail how this could arise for carbon removal in a key article entitled ‘Climate change is a waste management problem’, stating that ‘carbon dioxide is a waste product; dumping it into the open air is a form of littering’ [16]. As Lackner and Jospe point out, a shift to a waste paradigm provides the policy rationale for promoting CDR by ‘articulating carbon dioxide disposal as a public good, like sewage disposal or even national defence and public health’ [16]. Waste disposal in particular also recognizes the scale and nature of the carbon dioxide challenge—a tremendous amount of matter must be transferred into a place where it can be secured for thousands of years.

However, the analogy of CDR as waste management also has its dangers. In the way the ‘circular economy’ or ‘CO_2_ recycling’ is conceptualized by some fossil fuel actors, waste is no longer a by-product, but an opportunity, making it part of a resource frontier (see [9]). Valuing the waste normalizes production of it. Simply treating CO_2_ as a substance that needs to be recovered in order to be turned into value could actually draw focus away from the massive scale of the permanent storage required. A waste-to-value focus could also gloss over the fact that carbon pollution is a disaster with particular geographies and lived experiences, as well as global and intergenerational impacts. Some may view this neutralizing perspective as a feature of the frame, not a bug: as Lackner and Jospe write, the focus on emissions reductions has included a moral judgement against emitters, making everyone carbon sinners. However, ‘a waste management perspective makes it unnecessary to demonize or outlaw activities that create waste streams. It's okay for people to use toilets and generate garbage; society in turn provides appropriate means of waste disposal to protect the common good’ [16]. In this more neutral view, CO_2_ emissions are ‘a metabolic by-product of industrial activities on which billions of people depend to survive and thrive’, and now we must learn to safely dispose of them. Another feature of this view is that waste management does not demand a global transformation of energy infrastructure, Lackner and Jospe point out, but the construction of a parallel one, meaning that it does not threaten fossil industry interests and trigger opposition from them [16]. This too is viewed by Lackner and Jospe as a feature rather than a bug. There are a few problems with this level of simplification, though: (1) some people are profiting from these activities more than others and (2) a waste management/carbon capture and use paradigm can imply that emissions will continue to be generated, managed and moved through the system, rather than eliminated, as some actors may prefer. From another perspective, treating CO_2_ as waste is an admission of failure—failure to prevent the waste in the first place by moving expediently towards clean technologies.

So the analogy is nuanced, and not straightforward. Carbon removal as waste management may deserve uptake, both conceptually and in practice. But before embarking wholeheartedly on waste management regimes for the air, or for particular elements in the periodic table, we should look back at the history of solid and liquid waste management thus far—especially given that both of these techniques and institutions still face serious global challenges. How was it that waste in these other spheres came to be regulated? In accounts from the environmental history of waste, both cultural norms and industrial demands played a role. Examining lessons from the past can help us understand what thinking about CO_2_ as waste can do for us, and where adopting this paradigm might lead us astray.

## 2. Evolution of liquid, solid and gaseous waste regimes

Since the Industrial Revolution, societies have stepped up efforts to treat, regulate and recycle solid and liquid waste. Gases, however, have been treated as air pollution, but not exactly as ‘waste’, in the sense of developing regimes to manage their permanent disposal or reuse. Outdoor air pollution became an issue with the development of cities, with Romans complaining about the *gravioris caeli* (‘heavy heaven’) and *infamis aer* (‘infamous air’) over their city [17, p. 145]. However, the term ‘air pollution’ did not really emerge until the 1930s [18], with air pollution previously understood as the ‘smoke problem’. Discussions about smoke, particularly coal smoke, arose in the 1880s [19]. Reformers from civil society worked on smoke abatement for decades, and gradually new technologies were implemented, with air pollution finally becoming a national issue in the middle of the twentieth century. Legislation like Britian's Clean Air Act of 1956 and the US Clean Air Act of 1970 finally addressed air pollution on the national scale, and were followed by global-scale efforts in the 1970s and 1980s, such as the Convention on Long-Range Transboundary Pollution and the Montreal Protocol [17]. While progress is terribly uneven, some of the best examples of cooperation and progress on environmental issues come from improvements in air quality. Yet these are framed as lessons on dealing with pollution, rather than waste management—a subtle difference, but one worth exploring.

Why haven't gases been treated as waste? Gas emission control technology to capture and manage gases like CO_2_, CO, SO_2_, H_2_S, NO_X_ and H exists. Gas volumes are vast while the associated solids from their waste streams are much smaller, though thus far, the mass of the waste streams have not posed disposal issues. Moreover, these developments are largely within the industrial sector and the oil and gas industry, rather than something of broader public and cultural interest. In short, the average person does not have direct experience of managing gaseous waste the way they do with taking out their trash or flushing their toilet, meaning that there is not much of a cultural history of gaseous waste management yet. However, a rising interest in carbon capture and methane and use, new technologies such as air-to-fuels, and an increase in public discourse and regulation around natural gas flaring may change this.

In this section we will explore: How did waste management for solid and liquid forms of waste develop? What lessons or pitfalls can be learned from these realms and applied to an emergent gaseous waste management regime?

### 2.1. Liquid waste

When it comes to liquid waste, supplying water and disposing of water are necessarily intertwined: what comes into the home or factory must go out. Water supply systems were built before water carriage or sewer systems. By 1880, one-third of homes had water closets, and *per capita* water consumption jumped from 2–3 gallons per day to 50–100 gallons per day [20, p. 114; 21, p. 74]. The drive to build up water supply systems came not just from urban household demand but from industry, which built infrastructure like steam pumps, canals and water towers [22]. Part of this was due to the *needs* of industry for clean water; part of it was due to the provisioning being an opportunity *for* new companies in water provision. Initially, in the mid-1800s, private companies undertook much of the infrastructure for cities like France or London. From 1820 to 1880, urban population growth and the adoption of flush toilets drove the breakdown of the previous system of cesspools and privy vaults in the USA, which could not cope with the increase in piped-in water without building sewers to carry it away [23]. While the conflict between private and public *provision* is still playing out, it is worth noting that water carriage and wastewater *treatment* necessarily became a public service in many places. Centralized public works for water treatment became necessary, and it became a municipal rather than private responsibility because of both the health implications and the capital requirements [23].

Cultural drivers were important in constructing wastewater systems. The sanitary movement—notably, it was a social *movement*, encompassing both solid and liquid waste—arose in Great Britain in the 1840s and 1850s, led by elites and professionals with the aim of getting people to change their ideas about cleanliness habits, along with promoting urban public works for health goals [23]. The profession of the ‘sanitary engineer’, who was something of an environmental generalist and part of a profession that considered public health as well as engineering, was important in merging engineering and health [20, p. 201]. Many regulative, cognitive and normative shifts preceded the change from cesspools to sewage technologies [24]. In a study of the shift in The Netherlands, Geels explains how hygienist doctors and engineers worked in a coalition: the hygienists articulated problems and general solutions and the engineers offered technical designs [24, p. 1075]. There was also ‘issue linkage’, where waste was linked to social issues like poverty and class struggles [24]. Value change was also important: the sanitation movement facilitated the acceptance of sewage technology (and the costs of the capital-intensive system) by propagating the filth theory of disease, and persuading urbanites that it could improve health [20, p. 210].

Building systems to carry wastewater away was not a seamless or easy transition; it was much debated. Opponents of water carriage technology argued that there would be health hazards because of leakage and subsoil contamination, that drinking-water supplies and shellfish would be under threat from pollution, and that sewers would generate gases that would bring disease [23]. Moreover, there were concerns about the heavy tax burden—if financed with bonds, this would impose costs on future generations with no voice—and the way that water carriage would waste resources in human excreta that could be used for fertilizer [23]. Many of these would be familiar lines of debate when it comes to building carbon dioxide disposal infrastructure today. Sewage *treatment*, however, did not come for several more decades, with demands for legislation to protect water quality coming along with the Progressive movement for natural resource conservation more broadly [23]. Moreover, as Joel Tarr notes, ‘It is one of the greatest ironies in the history of technology and its relationship to the environment that a technology designed to improve local health conditions and eliminate nuisances—water-carriage technology or sewerage—had extremely devastating effects on both the environment and human health’ [20, p. 104].

### 2.2. Solid waste

Solid waste management also began in cities. From the 1770s to the 1860s, efforts to clean up cities went in tandem with excreta recovery, for agriculture and for industry [10, p. 199]. During the late eighteenth century, waste was collected from the cities with the idea of returning food as fertilizer to the countryside across Europe and North America [10]. Waste materials were also used in industry, such as vegetable rags used for papermaking, and animal bones used for manufacturing things like grease and glue. Butchering by-products were used to make all kinds of things, from matches to sugar refining to gelatin for photographic negatives. Large waste removal companies profited from emptying cesspools and from producing fertilizer, which in fact made them a vested interest that opposed sewer systems [10, p. 208]. In general, the early industrial period had a system of recycling that involved collectors who converted urban trash into raw materials, and purchasers who could extract useful elements from these new commodities and remanufacture new products with them [25, p. 72].

Municipal waste collection started to be organized during the turn of the century, with women's clubs who took on sanitation issues promoting this as part of the broader aforementioned sanitation movement [25, p. 121]. Sanitary reform had two factions—sanitary engineers, a technically elite group, and citizens' organizations, who never joined in a broadly based movement [21, p. 87]. As with liquid waste, there was some resistance and questioning of the role of government—but in general, cities adopted the view that government was responsible for public health and that refuse was part of the responsibility. Landowners and merchants, who already paid for private collection, rejected higher property taxes to pay for sanitation, but by 1900, municipal waste collection did become established in most US cities [25, p. 123].

Yet by the turn of the century, waste was no longer viewed as a resource, but as a burden: disposal became separated from production [25, pp. 14–15]. Technology played a role here: the development of synthetic fertilizer via the Haber–Bosch process meant that urban by-products were devalued as fertilizer. Recycling and waste management also became a much more formal industry. The first half of the twentieth century also saw the creation of the organized waste trade, with a National Association of Waste Material Dealers founded in 1913, and a membership that had grown to 450 by 1928 [25, p. 118]. With this formalization, and new specifications and subdivisions to manage industrial waste, the independent wastepickers and ragmen who salvaged small amounts were less relevant [25].

World War II involved scrap drives to conserve materials, but neither Depression-induced thrift nor World War II campaigns brought recycling back [25, p. 259]. Part of this was because automation and industrialization lowered the cost of raw materials; but an emergent ethos of consumerism was also a factor. Consumerism was not questioned until the 1960s and 1970s, when recycling became a cultural value again. Yet the recycling that emerged in the 1980s and 1990s as a result was quite different from the earlier system, as Strasser points out: households put out refuse with the expectation that it will be re-used, but they are not participating in a two-way exchange of materials [25, p. 286]. Moreover, despite the renewed interest in recycling from the 1970s until today, recycling has only had mixed success. China's ‘Operation National Sword’, announced in 2017, in which China has banned imports of recovered mixed paper, recycled plastic, scrap metal, and other waste streams [26], made apparent the flaws of a global recycling system in which goods declared to be waste in the Global North become recovered with cheaper labour in the Global South.

### 2.3. Lessons for gaseous waste

What drove the development of waste treatment and recycling in solid and liquid waste, and would the same drivers apply today? First, culture played a critical role, but the needs of and opportunities for industry were also important at different stages—and this is worthwhile for both entrepreneurs and climate activists to keep in mind when thinking about carbon removal or drawdown. Value-change and the development of new water carriage technologies are a self-reinforcing loop, according to Tarr *et al.* [23]: developing the technology actually created and reinforced other values, such as a belief in the need for planning, expertise and bureaucracy, as well as a regulatory role for the state. In short, the success in developing and deploying the technology strengthened those values. This highlights something about the pernicious effects of possible ‘mitigation deterrence’ effects of developing negative emissions (see [27]). That is, if mitigation is deterred by CCUS, alleviating the damage might not be as simple as a policy course correction—the effects of the mitigation deterrence may have a long afterlife in the form of eroding this loop, contributing to a corrosive mistrust. An implementation of carbon removal that fails to deliver measurable removals—due to poor life cycle analysis, lax policy that allows large residual emissions, or general overpromising—risks decreasing belief in the capacity of the state or in the role of experts in managing climate change.

A second takeaway is that when it comes to solid waste, there has been an ebb and flow between systems of reuse and systems of disposal, with solid waste recycling currently in crisis. If carbon management follows the current paradigm of solid waste, where consumers are made to feel as if they are recycling and putting in adequate effort (i.e. by diligently purchasing carbon removal credits with flights), but the lion's share of waste is simply discarded, that will also be corrosive to the self-reinforcing loop between value-change and the development of new technologies. Looking at the way views of solid waste have changed, as well as the failure to truly recycle many solid materials, brings up questions of how difficult it will be to actually revalue CO_2_ as waste. Previously, cheap fossil fuels and their lobbyists have stymied attempts to build a circular economy. In the next section, we will turn to one particular movement that attempted to revalue waste under an umbrella term—chemurgy—to explore whether ‘carbon removal’ might be a similarly short-lived umbrella term.

## 3. Opportunity lost: chemurgy and the failed movement for a circular economy in the 1930s

In 1935, three hundred notable scientists and industrialists came together in Dearborn, Michigan, and signed the ‘Declaration of Dependence upon the Soil and the Right of Self-Maintenance’. It was written upon hemp paper, under a flag made from hemp. The ceremony was performed in a replica of Independence Hall, with Thomas Jefferson's desk and Abe Lincoln's table shipped in for the signing. Outside, along the riverbanks, small water-powered factories manufactured automobile sub-assemblies using clean energy and green materials, and processed soya beans into plastic parts. The conference aimed to articulate a vision of a green supply chain and a farm-based car, as well as a decentralized industry rooted in agriculture that would enliven rural areas and promote national self-sufficiency [28, p. 14]. It was a vision that would be at home in today's discourses around a new carbon economy, the circular bioeconomy, regenerative agriculture and the Green New Deal.

The follow-up conference in 1937 brought Henry Ford and George Washington Carver together to discuss their shared dream of chemurgy: engineering new uses for plants. The term chemurgy first appeared in print in 1934, a portmanteau of *chemi* *+* *ergon* (work). Chemurgy had three goals: to develop new non-food uses of crops, to substitute industrial crop for surplus commodities and to find profitable uses for various agricultural wastes and residues [29]. But to some of its promoters, it offered a dream of universal abundance: an ‘organized attempt to create true wealth’, wrote journalist Christy Borth, ‘that only real wealth which lies dormant and neglected in the powers of the soil and the air and the sun and the mighty minds of people’, which could be channelled into a more abundant life for all [30].

There are a few reasons why chemurgy arose at just this moment in history. Part of it involved socio-economic troubles, such as large farm surpluses. The New Deal approach was to reduce surpluses by paying farmers to plant less, which chemurgists opposed stridently as a form of waste. Rather, the chemurgists suggested that surplus farm materials should be used to make new non-food products. There was an idea that agriculture was ‘out of balance’, which extended to ideas about an imbalance between rural and urban, or between agriculture and industry. Chemurgy was seen as a socio-technical solution to these imbalances. There are also technological reasons for the emergence of chemurgy in the 1930s: as chemurgy promoter Wheeler McMillen pointed out, the tools for chemurgic performance ‘have just recently come into view’; those being the science of organic chemistry, the science of plant genetics and the ‘art of the engineer’ [31, p. vii]. Putting a pound of food on the market, explained McMillen, required growing another pound of inedible material—straw and cornstalks; forms of cellulose, protein, oils, starches and sugars. Cornstalks are light, bulky and widely scattered—but new devices for chopping and baling cornstalks made it possible to think about processing them. In short, the whole socio-technical system around mechanized agriculture, genetics and chemistry made the chemurgic dream possible. It seemed like the technologies to build a circular economy—as we might call it today—were now at hand.

The movement was influenced by three diverse lineages, according to agricultural historian Randall Beeman. The first was an ‘agrarian-economic’ vision of rural development. Chemurgy was seen as a way to make depressed rural regions strong, which was especially prevalent in the southern USA. Second, there was a ‘corporate-technocratic’ vision, which envisioned a society, and agriculture, guided by ‘scientists in the service of industry’, stripping the culture out of agriculture and imagining large farm units staffed by managers, scientists and workers [32, p. 26]. This was not necessarily imagined to be an easy transition by those writing at the time, and as one scholar writing in 1940 described, family farms would disappear and consolidation would take place so that large units would have the capital for heavy machinery. ‘This implies some unpleasant things. It suggests that the farmer will be faced with the insecurity, the loss of control over his own life, the regimentation of the present-day factory worker’ [33]. Indeed, this is the backdrop on which chemurgy took place, which conflicted at times with the first lineage. As Frank Uekoetter writes, chemurgy sought to empower chemists, and had a vision of expert rule, where chemists would serve as ‘supreme economic coordinators’ [34]. The third vision of chemurgy was, in Beeman's terms, a ‘conservation-ecological’ strain of thought, which highlighted renewability and balance. Petroleum reserves were only imagined to last a matter of decades, and ‘agrol’, or ethanol fuel, seemed destined for success. Chemurgy was a noble ‘attempt to put man on a pay-as-you-go basis in terms of raw materials beginning now, before he must, the policy of living on his annual income of growing things and using less of the stored capital of mineral wealth that nature accumulated through millions of years’ (Borth, in [32]).

Chemurgy, as a start-up scientific movement, did achieve some successes. Lobbying efforts by the chemurgists got the USDA to fund four new national research laboratories, in 1938, and a survey afterwards showed 10 000 research projects and 1300 institutions interested in chemurgical research [35]—not bad for a brand-new field. Chemurgists also achieved some strides towards American self-reliance, setting up an American flax paper industry and growing the southern pine industry [31, p. 25]. However, the energy of these great industrialists and scientists working in concert did not translate into a new paradigm. Despite some initial successes, by the 1950s, the term chemurgy had already largely fallen out of favour. The simplest explanation for this is that petroleum was a cheaper material for fuel and other goods [36]. From a sustainability standpoint, of course, petroleum was inferior. Chemist William Hale saw ethanol as a far superior fuel to petroleum, and he viewed developing it as a moral right, lamenting how vested interests were obstructing progress.

At this writing our petroleum corporations are face to face with conditions not unlike those which confronted railway corporations some twenty years ago, when they opposed with all vigor every attempt of automobile and bus to enter the transportation field. … Though some may consider a motor operating on ethyl alcohol and water as revolutionary, it is not any more revolutionary to poorly powered motors of today than was the automobile to the inefficiently powered railway trains of yesterday. The time is opportune for petroleum corporations to wake up, rub the cobwebs from their eyes, and listen to reason. Alarm signals are already at the gates of refineries and they keep on ringing: ‘Remember the Railways’. [37, p. 129]

But there are several other important factors for why chemurgy never took off—some of which are germane to hopes for a carbon-to-value today. There were inherent problems with the movement: as Beeman details, it promised too much to too many, it disappointed people who got excited about it, and its emphasis on rapid technological change was out of step with the pace of farmer adoption [32, p. 42]. Chemurgy was a long-term programme of research. For farmers, the goal of national self-sufficiency was not always appealing, and the New Deal offered immediate subsidies. Chemurgical projects often had a wide gulf between pilot projects or prototypes and full-scale production. Moreover, many agricultural industries are not lucrative for investors, because they have downtimes in between harvests and only have raw materials available once a year, making progress slow [29, p. 98].

There were also passive, extraneous factors working against chemurgy: the war took away the focus and resources from chemurgic research. There was also active opposition. ‘The chemurgy movement was under attack from an unholy alliance of New Deal politicians, university scientists, atheists, the oil companies, bankers, free traders, the Roosevelt administration, large farmers, and the synthetic chemical industry’, Skrabec explains [28]. Moreover, there were internal challenges, like reliance on private funders and personality conflicts within movement leadership. In Beeman's assessment, chemurgy was guided by corporate-technocratic scientists in the employment of industry, rather than by agrarian leaders [32, p. 32]. Uekoetter, too, sees chemurgy as not being about the advantages of renewable energy over non-renewable sources, nor even about monetary interests, but about a professional creed [34], one that saw chemistry expertise as the foundation of a new age.

The technocratic reapportionment of nature's resources also had dark undertones—and while these did not necessarily lead to its downfall, they would have likely prevented its success. Prominent chemurgist William Hale praised Germany for building anew upon a ‘strictly scientific basis’, and he saw Germany, Italy and Japan as ‘scientifically organized’ nations which should have access to more land, with large apportionments stretching from temperate to tropical zones [37, p. 211]. This sounds like a ringing endorsement of colonialism—and in a sense it is—though Hale also critiqued colonialism for creating a situation where nations who had previously seized lands now controlled them without developing them. ‘Prior to the chemical age there was little need for any great nation to possess tropical lands, but in the coming of chemurgy it is absolutely necessary that every great nation cultivate certain of its requirements on super-sunlit land. In sunlight is National Security’, he wrote. Hale's [38] 1952 book, *Chemivision*, explicitly begins to mix race and religion, with a foreword by Howard B. Rand, who headed the Anglo-Saxon Federation of America. It describes organizing the Earth into four ‘Sations’, or self-sufficient mega-regions (sorted by race), under which each group would be self-sufficient, and at which point they would not think about communism, either [38].

*Chemivision* appeared at the trailing end of chemurgy, and as its dreamers and promoters aged, the movement seemed to simply go off the rails into incoherence. Yet chemurgy did not completely vanish—it had a small uptick of interest in the 1970s, coinciding with the energy crisis and environmental concerns. The 1970s version of chemurgy was more about using waste for industrial profit. A 1970 conference of the Chemurgic Council was entitled ‘Chemurgy—for Better Environment and Profits’, where a noted chemist Robert Cairns exemplified the tone of the day: ‘If we are to continue to live in comfort in a closed environment such as that afforded us on the planet earth, we've got to pay increasing attention to the recycling of used goods and materials’ [39]. The final conference before the Chemurgic Council disbanded was held in 1973, entitled ‘New Resources from the Sun’. However, then oil prices went down again.

Will carbon removal and ‘negative emissions’ be a similarly short-lived movement—a relic of the early twenty-first century? Or will research in this field mature into a genuine discipline of carbon management? We can draw a few lessons from the example of chemurgy, for both biomass-based carbon recycling as well as for mitigation. First, the case of chemurgy shows why hybrid forms of carbon removal involving agriculture are still a tough proposition today. Many of the factors plaguing chemurgy's prospects—the difficulty of crossing from prototype to commercial scale, the difficulties inherent in making investor-grade profits from agriculture, the potentially low interest from farmers—would still be in play.

Second, the way chemurgy was driven by industry people and technocrats working on grander scales mirrors the essential problem with using crops for carbon removal, as well as CDR more broadly: the concept is driven by scientists and entrepreneurs, not the people who are working in or even planning the fields. As Uekoetter notes, chemurgy's ‘fuel alcohol project was essentially a vehicle for a grand expertocratic vision with some technological work attached’; while there was something of an audience for this, there was not a following or a political coalition [34].

Third, inexpensive petroleum and its lobbyists precluded the development of new technologies for fuel and other bioproducts. Today's push for CO_2_-EOR might similarly pre-empt more technically elegant solutions (defined here as non-extractive, simpler and easily scalable beyond particular geographies), whether they be advanced biofuels, air-to-fuels, or other circular carbon technologies. As Albert Lin has cautioned, sinking costs into a particular CDR approach at an early stage can risk locking in particular technologies [40].

More broadly, the tale of chemurgy can also be read as a cautionary one regarding hopes that the crises of the Anthropocene might lead to an embrace of science, rationality and planning. In chemurgy, despite the nationalism expressed by the movement, one can also discern the chemurgists' hopes for crisis to drive a broader form of rationality, for order to emerge out of the disorder of depression and then world war. As Hale wrote, ‘Suppression of chemurgy by gold worshippers and false economists, lest agriculture become supreme, smacks of the Dark Ages. Perhaps a new Dark Age is in the making. … Possibly future generations will look upon the Second World War as instrumental to the reign of science’. It is easy to rewrite these kind of hopes with the Anthropocene in mind—there are echoes of this hope in some of the literature positing the crisis of the Anthropocene a turning point for embracing more rational, sustainable stewardship grounded in science. The darker moments of the twentieth century, accompanied by cheap oil, did not give rise to scientifically managed biofuels.

## 4. Conclusion: the future of gaseous waste management

Net-zero targets are now popular, following the guidance for ‘balancing sources and sinks’ in the Paris Agreement, and this idea of balance aligns easily with the circular economy. But the challenge here is that there are two different versions of net-zero: one where net-zero is a temporary phase on our way to ceasing fossil fuel extraction, and another where net-zero is a way to continue extracting fossil fuels for the foreseeable future. In the former, carbon dioxide production is phased out; in the latter, it is simply balanced with large amounts of removals.

One feature of waste is that it becomes a public responsibility, or hazard, *after* it is discarded. Before that, it is in a state of private ownership. It is the point of disposal when it crosses the margin—in earlier times, this was the boundary of the house. When waste moves into public space, it becomes a public matter; and once it is municipal solid waste, it is a civic concern for experts [25, pp. 7, 19]. Following this logic applied to fossil fuels, it is the point of combustion when they become a civic concern—the point of throwing away—not the point of production. This is a key weakness in the waste management view of carbon removal: it shifts political emphasis away from the point of production, at least in contemporary discourse.

This need not necessarily be the case. Ideas from circular-economy advocates who are rethinking production could provide an opening to address carbon production more directly. Perhaps the most productive way for scientists, policymakers and science communicators to engage with waste management frames for carbon removal is not simply to uncritically embrace nor reject these frames, but to contextualize them honestly within the struggles and goals of the larger circular economy project.

If anything, looking at the history of attitudes towards waste illustrates the limits of rhetoric and cultural change or norm shifts without real policy. Sanitation, though it employed many professional engineers, was a genuine movement. Carbon removal, if it is a movement, is more like chemurgy: a movement of technocrats and scientists, not women's organizations and civil reformers. This does not bode well for its success. We can see that the metaphor of carbon as waste is both useful and deceiving. It has not yet been inspiring, however, since awareness of the danger of climate change and the scale of emissions is not yet high enough for action (with the exception of Project Drawdown, a civil society project on decarbonization approaches informed by science yet not well represented in the scientific literature). Another lesson from history is that up until the ecological movement, civic reformers were able to work with regulators and industry to some degree—for example, on smoke abatement, at times helping industry find technical solutions. But as Uekoetter highlights, after the environmental movement, the dominant pseudocorporatist approach broke down, with no communication or trust between industry, officials and publics [19, p. 243]. That breakdown of trust is even worse today than in 1970, and it will certainly affect carbon removal politics.

If this interaction between values and technology is a self-reinforcing loop, as posited by Tarr *et al*. [23], where social values like those of sanitary campaigners encourage development of new technologies and feedback from the successful technology reinforces the original values, this presents some challenges when applied to carbon management. First, intense carbon management in one region or country may not make any appreciable difference in the global climate system, so there is a collective action problem. This highlights how important designing policy for economic co-benefits, including jobs in carbon clean-up, will be. Second, the social values present in the late eighteenth and mid-nineteenth centuries, when liquid and solid waste management regimes developed, have shifted. They were developed in a time with a strong belief in modernity. But a key takeaway from Joel Tarr's work is that policies to manage pollution in one domain—air, land or sea—often resulted in the transfer of contaminants to another less regulated medium, and other technologies get drawn in to deal with those negative impacts, ‘thereby involving the society in loops of retrofits and technological fixes’. This is one of many factors contributing to a decreased faith in technological progress. What happens to the notion of carbon management when that belief in progress and modernization has shriveled? Hopefully the logic of the self-reinforcing loop works both ways: developing carbon removal technologies can inspire social values around decarbonization and climate action for the public good, reinforcing the demand for still better technologies and stronger social values. The sanitary movement also provides an illustration of how emphasizing public health can generate a movement that can argue for bearing the costs of developing infrastructure to clean up waste, as sanitary reformers did. In a time of increased sensitivity and public responsibility to collective public health, the successes and challenges of the sanitary movement are well worth revisiting.

## Data accessibility

This article has no additional data.
